# In Vitro Production of Somaclones with Decreased Erucic Acid Content in Indian Mustard [*Brassica juncea* (Linn.) Czern&Coss]

**DOI:** 10.3390/plants10071297

**Published:** 2021-06-25

**Authors:** Chitralekha Shyam, Manoj Kumar Tripathi, Sushma Tiwari, Niraj Tripathi, Ravindra Singh Solanki, Swapnil Sapre, Ashok Ahuja, Sharad Tiwari

**Affiliations:** 1 Department of Genetics & Plant Breeding, College of Agriculture, RVS Agriculture University, Gwalior 474002, India; shyamchitralekha@gmail.com; 2 Department of Plant Molecular Biology & Biotechnology, College of Agriculture, RVS Agriculture University, Gwalior 474002, India; sushma2540@gmail.com (S.T.); ravindrasolankijnkvv@gmail.com (R.S.S.); ahujarrljm@gmail.com (A.A.); 3 Directorate of Research Services, JN Agriculture University, Jabalpur 482004, India; nirajtripathi@jnkvv.org; 4 Biotechnology Centre, JN Agriculture University, Jabalpur 482004, India; swapnil.spr@gmail.com (S.S.); stiwari@jnkvv.org (S.T.)

**Keywords:** Indian mustard, erucic acid, callus culture, cell suspension culture, somaclones

## Abstract

*Brassica juncea* is a crucial cultivated mustard species and principal oilseed crop of India and Madhya Pradesh, grown for diverse vegetables, condiments, and oilseeds. Somaclonal variation was explored as a probable source of additional variability for the manipulation of fatty acids, especially low erucic acid contents that may be valuable for this commercially important plant species. The plantlets regenerated from tissue cultures (R_0_), their R_1_ generation and respective parental lines were compared for morpho-physiological traits and fatty acid profile for the probable existence of somaclonal variations. The first putative somaclone derived from genotype CS54 contained 5.48% and 5.52% erucic acid in R_0_ and R_1_ regenerants, respectively, compared to the mother plant (41.36%). In comparison, the second somaclone acquired from PM30 exhibited a complete absence of erucic acid corresponding to its mother plant (1.07%). These putative somaclones present a source of variation for exploitation in the development of future mustard crops with low erucic acid content.

## 1. Introduction

Indian mustard (*Brassica juncea* L.) displays an immense polymorphism and is the basis of diverse types of vegetables, condiments, and oilseeds. It is an important oilseed crop globally, nutritionally affluent in fat, carbohydrate, vitamins, minerals, and water. As a result, its oil is expansively exercised in the medicinal, cosmetic and leather industries. It is an oilseed in high demand proving an increasing trend by fulfilling demand and supply with desired qualitative traits. The occurrence of erucic acid (C22:1) in elevated concentration (35–50%) is a significant drawback to mustard oil [1,2,3,4,5]. This high concentration of erucic acid in edible mustard oil makes it nutritionally unfavorable for the human diet. This is because of its role as a causative agent of a disorder of lipid metabolism called lipidosis, myocardial infarction, and increasing blood cholesterol [6,7,8,9,10]. It has also been reported as a causative agent of lipidosis in children. In addition, such oil properties are also serviceable for industrial purposes [11]. There is an urgent need to reduce the erucic acid content, and employing different biotechnological advancements, including somaclonal variation, could solve this dilemma.

The exploitation of somaclonal variation has endeavored to improve this crop in recent years. In vitro selection using the somaclonal variation method offers a prospect for the rapid and comprehensive creation of functional mutants or somaclones tolerant/resistant to some biotic and abiotic stresses and other qualitative traits. Such plants can be an outstanding contributor to the tolerant/resistant gene(s) in further exploitation in conventional and/or molecular breeding programs. In vitro selection employing pathotoxins as a selection mediator for *Phoma lingam* resistance has been documented in *Brassica napus* [12]. Anuradha et al. [13] evaluated comparatively among somaclonal, EMS, and gamma-ray raised changes in *Brassica juncea*. A somaclone BIO-902, derived from the mustard variety Varuna, has been notified as a new variety that holds resistance to shattering, including higher yield by Katiyar and Chopra [14]. Ahmad [15] attempted in vitro selection in *Brassica* for salt tolerance. In vitro selection for salt tolerance and agronomical traits was also tried by Jain et al. [16]. Roy and Saha [17] identified three low erucic acid-containing genotypes of Indian mustard from F1 doubled haploid, generated using another culture. Subsequently, Iqbal et al. [18] cultured ovules obtained from inter specific crosses of *B. napus* and *B. junciea* for fatty acid manipulation. Apart from in vitro techniques, two genetic engineering approaches were made by Sinha et al. [19] for engineering *B. junciea* with low erucic acid content. A novel FatB thioesterase obtained from *Diploknema butyracea* was transferred into the *B. juncea* crop in the first approach. In the second approach, the *B. juncea* fatty acid elongase was restricted at the genetic level by incorporating hairpin RNA to cause post-transcriptional gene silencing. Novel FatB genes were also cloned and characterized by Jha et al. [20] to manipulate vegetable fats/oils of commercial importance in *Brassica species*. Nevertheless, except for a few, the contribution of in vitro selection in conjunction with somaclonal variation procedures in the breeding program in *Brassica juncea* is narrow [16], especially for erucic acid. Nonetheless, encouraging reports have been evidenced in wheat and barley [21], soybean [22,23,24,25], groundnut [26,27,28], onion [29], and *Withania somnifera* [30] with variable degrees of successes.

In the current investigation, an effort has been made to investigate the existence of somaclonal variation for low erucic acid content that can further be utilized as a source of added variability for the improvement of Indian mustard. To accomplish this, two genotypes viz., CS54 and PM30, were selected for the present research based on erucic acid content, whether higher or low, with two explants viz., immature cotyledons and seeds, to establish callus and subsequently to raise cell suspension cultures followed by an efficient and reproducible plantlet regeneration. However, the selection of genotypes with low erucic acid content with higher yield is hampered by various aspects, including plant species, genotypes, explant sources, nutritional requirements, and environmental behavior. Thus, the procedure and conditions for callus induction and shoot differentiation followed by plantlet regeneration were optimized for generating somaclones via callus and cell suspension cultures.

## 2. Results and Discussion

The potential and limitations of the tissue culture technique for creating new genotype(s) with desired characteristics of agricultural importance have been documented in a considerable number of crop plants, including Brassica. Attempts to select lines with low and high erucic acid content are based on the assumption that depending on the cultivation protocol, the level and content of specific metabolites in tissue culture can be influenced to some extent. Certain metabolic pathways of a particular plant species lead to the synthesis of a specific metabolite (i.e., erucic acid). Their production also operates in cells and cell lines selected based on particular metabolite yield, and these lines were derived from the regenerant.In contrast, regenerants derived from these lines may have altered levels of certain metabolites of interest. 

### 2.1. Callus and Cell Suspension Cultures

**Figure 1:**
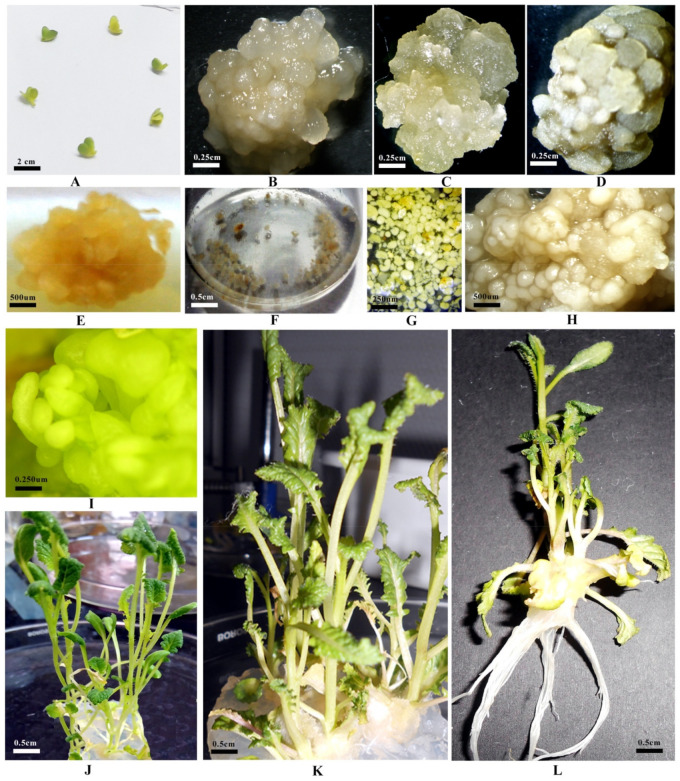
In vitro morphogenesis and plant regeneration from callus and cell suspension cultures in Indian mustard: (**A**) Cultured immature cotyledons after 10–14 days in culture; (**B**) Callus induction from cultured immature cotyledons of genotype CS54; (**C**) Callus induction from cultured immature cotyledons of genotype PM30; (**D**) Callus induction from cultured immature seeds of genotype CS54; (**E**) Callus induction from cultured immature seeds of genotype PM30; (**F**) Raising of cell suspension from immature cotyledons and seeds at the same time in a liquid medium; (**G**) Initiation of cell clumps and embryoid formation derived from embryogenic calli of immature cotyledons and seeds; (**H**) Globular stage somatic embryoid formation; (**I**) Germination of embryoid; (**J**) Multiple shoot formation from callus cultures of genotype CS54; (**K**) Multiple shoot formation from callus cultures of genotype PM30 and (**L**) Rooted regenerant.

In the current investigation, for raising callus cultures, Murashige and Skoog (MS) medium amended with 3.0 mg L^–1^ 2,4 dichlorophenoxyacitic acid(2,4-D) performed superior among all the combinations tested (Table 1 and Table 2; Figure 1A–E) from cultured immature cotyledons and seeds. Earlier, Shyam et al. [31], Akmal et al. [32], and Lone et al. [33] also documented maximum callus induction with the application of 2,4-D in a concentration of 2.0–2.5 mg L^−1^. Subsequently, the reports of Alam et al. [34] and Nasrin et al. [35] were in agreement with the present investigation that the nature and color of callus were significantly influenced by concentrations of exogenous levels of 2,4-D. Similar results have also been obtained by Mishra et al. [25] in soybean.

Embryogenic calli were transferred to liquid media and were agitated mechanically to obtain cell clumps or embryoids. Friable calli, when agitated, were straightforwardly broken and separated into clumps of ~2.0–6.0 mm sizes (Figure 1F). Later, agitation splinted these clumps into small cell aggregates and embryoid formation (Figure 1G,H). The present results align with the earlier observations of Akmal et al. [32], who also documented globular and heart-shaped embryoid development in a liquid medium. Liquid medium supplemented with 3.0 mg L^−1^ 2,4-D in combination with 0.5 mg L^−1^ 6-Benzylaminopurine(BAP) facilitated higher growth rates (Table 3), this is in accordance with the reports of Mishra et al. [25] for soybean, Shyam et al. [31] for Indian mustard, Uikey et al. [36] for *Rauwolfia serpentina*, and Tripathi et al. [37] for sandalwood cell suspension cultures, as they recorded the highest growth rate of embryogenic tissues with the application of 2.0–3.0 mg L^−1^ 2,4-D in combination with 0.5 mg L^−1^ BAP.

**Figure 2:**
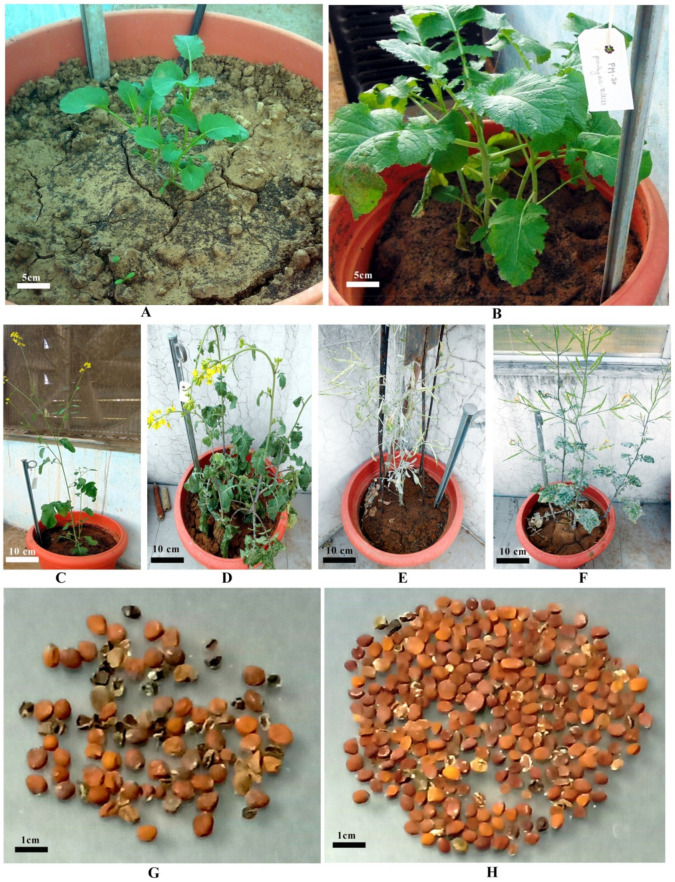
Hardening of putative somaclones and production of seeds:(**A**) Regenerant of putative somaclone CS54 transferred into a greenhouse for hardening; (**B**) Regenerant of putative somaclone PM30 transferred into a greenhouse for hardening; (**C**) Regenerant of putative somaclone CS54 transferred into a polyhouse for hardening at the flowering stage; (**D**) Regenerant of putative somaclone PM30 transferred into a polyhouse for hardening at the flowering stage; (**E**) Regenerant of putative somaclone CS54 transferred under field conditions at maturity stage; (**F**) Regenerant of putative somaclone PM30 transferred under field conditions at maturity stage. (**G**) Seeds of putative somaclone CS54 and (**H**) Seeds of putative somaclone PM30.

The response to callus induction applying 2,4-D was more pronounced in terms of type, nature, and color of callus proliferated. However, plant regeneration has not been obtained on a medium amended with 2,4-D alone from cultured immature seeds in optimum frequencies. Therefore, consecutively to augment shoot regeneration efficiency, callus cultures were subsequently inoculated on MS media fortified with BAP. Well growing callus cell lines of genotypes CS54 and PM30 were subjected to MS.5B (MS + 0.5 mg L^−1^ BAP) regeneration medium for shoot organogenesis (Table 1 and Table 2; Figure 1I–L). The present findings are supported by previous verdicts of Thakur et al. [38], Shyam et al. [31], Kumar and Srivastava [39], and Dhania and Singh [40] in mustard, Sharma et al. [41] in grape, Vibhute et al. [42] in *Citrus species*, and Mishra et al. [25] in soybean. However, in contrast to the present findings, multiple shoot formation in cotyledonary callus of Indian mustard (*Brassica juncea* cv. Prakash) was induced on modified MS media supplemented with a higher intensity of cytokinin viz., kinetin or zeatin, in combination with lower indole-3-acetic acid (IAA). Cytokinin BAP alone or in combination with auxins IAA or α-naphthalene acetic acid (NAA) did not support plantlet regeneration [16]. Plantlets acquired from cell lines of genotypes CS54 and PM30 were transferred under greenhouse conditions (Figure 2A,B), followed by polyhouse (Figure 2C,D) for hardening, before transferring under field conditions (Figure 2E,F), and the seeds were harvested after maturity (Figure 2G,H), and subsequently, the R_1_ generation was obtained from the growing R_o_ generation.

### 2.2. Morpho-Physiological Variations between Mother Plants, R_0_, and R_1_ Generations of Putative Somaclones

The data of different morpho-physiological traits are presented in Table 4. R_0_ and R_1_ generations of somaclones derived from cell lines of both the genotypes showed significantly reduced plant height, days to maturity, siliqua length and number(s) per plant, number(s) of seed per siliqua and plant, seed yield per plant, and biological yield when compared to mother plants. However, the number(s) of primary branches and days to 50% flowering were not significantly varied. The number(s) of secondary branches per plant increased in both putative somaclones significantly compared to the mother plant at a 5% probability level of significance. No statistically significant difference was recorded between the performance of R_0_ and R_1_ plants for different morphological parameters investigated except number(s) of silique per plant for somaclone derived from cell lines of genotype CS54 where R_1_ regenerants showed more number(s) of silique per plant as compared to R_0_ regenerants. Perhaps this happened due to the mutations of genes and induction of somaclonal variation and the activation of recessive genes that reduced expression of most morpho-physiological traits, as stated earlier by D’Amato [43]. They distinguished those variations that arise at the period of cell division and differentiation in vivo. Meristem cells that assist identical ‘germ lines’ are usually insusceptible to such genetic alterations. In the usual life span of a plant, the mutant somatic cells are eradicated throughout sexual reproduction and are not passed on to the progeny. Still, such mutant cells have an excellent chance to gulf and multiply (as do non-mutant cells) when plant tissues are subjected to the culture. Daunting selection burdens on cultured cells can result in privileged growth of mutant cells, creating mutant cell lines from which whole plants were regenerated. Jain et al. [16] also evaluated in vitro regenerated materials under field conditions to evaluate the somaclonal variation in R_1_ generation. Some of the plants showed significantly higher yield and/or other improved characteristics than the control. In addition, a dwarf plant type was also identified. Several plants were selected from this generation and carried forward to R_2_ generation, and most of these lines bred true in the R_2_ generation.

### 2.3. Fatty Acid Profiling of Putative Somaclones

**Figure 3:**
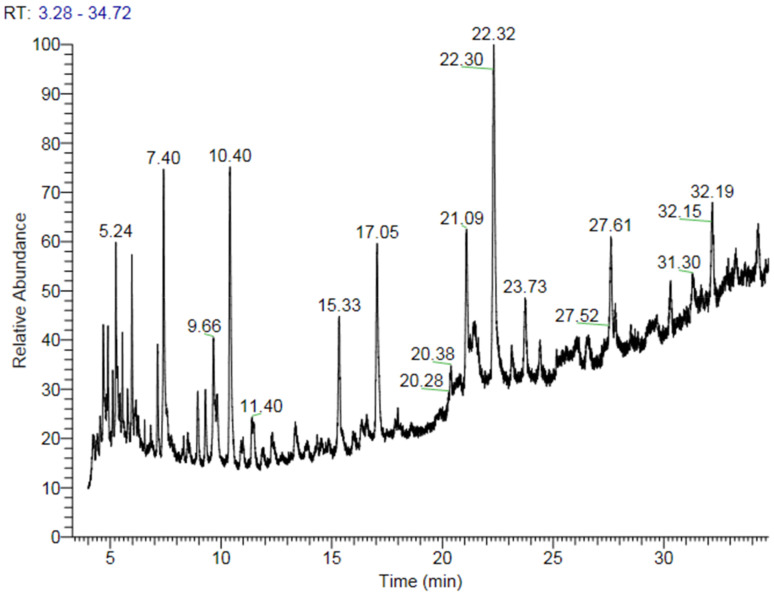
Gas chromatography-mass spectroscopy of somaclone regenerated from callus cultures of genotype CS54.

The fatty acid content and its profiling on putative CS54 somaclone revealed several saturated and unsaturated fatty acid variations. Thirty-nine fatty acid components were detected and classified into eleven groups depending upon RT (Retention Time). The percent value ranged between 2.88% and 10.90%. The highest content (10.90%) was recorded for Ethyl 9,12,15-octadecatrienoate, 9,12,15-Octadecatrienoic acid, methyl ester, (Z, Z, Z)-, and n-Propy1 9,12,15-octadecatrienoate at RT 10.4 on given conditions, whereas the lowest value (2.88%) was recorded for Ethyl 9, 12, 15-octadecatrienoate, 9, 12, 15-Octadecatrienoic acid, methyl ester, (Z, Z, Z)-, and n-Propyl 9,12,15-octadecatrienoate at 11.4 RT (Table 5; Figure 3). These fatty acids were further categorized for a particular one found in mustard oil, i.e., palmitic, oleic, linoleic, linolenic, or erucic acids (Table 6).When comparing erucic acid content and profile between the mother plant and R_0_ and R_1_ generations of putative somaclones regenerated from cell lines of genotypes CS54, it was interesting to note that plantlets regenerated in vitro, the erucic acid content recorded was 5.48% and 5.52%, respectively, in R0 and R1 regenerants as compared to mother plants (41.36%) of genotype CS54 (Table 6). However, palmitic and linolenic acids increased significantly in putative somaclones and R_1_ generation than in the mother plants. Moreover, no statistically significant difference was evidenced between R_0_ and R_1_ plantlets concerning different fatty acids.

**Figure 4:**
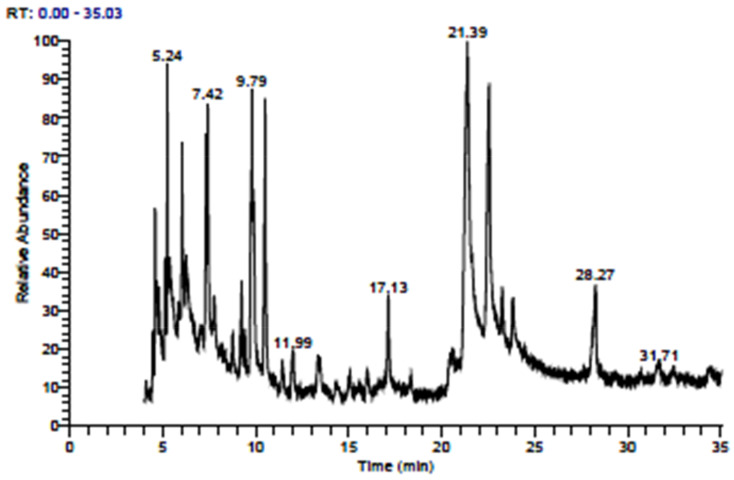
Gas chromatography-mass spectroscopy of somaclone regenerated from callus cultures of genotype PM30.

Fatty acid profile analysis of somaclones derived from callus cultures of genotype PM30 (Table 7 and Figure 4) also revealed variation in different fatty acid contents. A total of 27 fatty acid compounds were detected clustered into seven groups based on RT (retention time). The highest percentage (25.36%) was evidenced for Glycidyl oleate, 9-Octadecenoic acid (Z)-, 2-hydroxy-1-(hydroxymethyl) ethyl ester with 9-Octadecenoic acid, 1,2,3-propanetriyl ester, (E, E, E) eluted at 21.39 RT, while the lowest content (1.62%) was recorded for 1-Heptacosanol, Hexacosylpentafluoropropionate, Hexacosyl heptafluorobutyrate, 17-Pentatriacontene, Oleic acid, 3-(octadecyloxy) propyl ester, and Octadecane, 3-ethyl-5-(2-ethyl butyl) expressed at 11.99 RT. These fatty acids were further categorized for a particular acid found in mustard oil and presented in Table 8. The complete absence of erucic acid was detected in putative somaclone R_0_ and R_1_ generations regenerated from callus and cell suspension cultures of genotype PM30 despite its presence (1.07%) in the mother plant. Although palmitic and oleic acid ratios considerably increased in R0 and R_1_ generations of putative somaclones compared to mother plants. Conforming findings have also been reported by Jagannath et al. [44], who observed a decrease in erucic acid content, enhancing the oleic acid, giving a better ratio of linoleic and linolenic acids. Afterward, in Indian mustard oil, Singh et al. [5] reported that the trait high erucic acid content was partially dominant over low erucic acid content and emphasized that selection for low erucic acid would result in isolation of plants with high oleic and linoleic acids. Hence, it should be convincing to advance high oleic acid lines having low erucic acid content in mustard. A total of 11 double low lines were selected from a pool of 1200 lines in F_7_ generation by Priyamedha et al. [9]. Amongst the selected lines, two viz., BPRQ-2-1-5 and BPRQ-2-2-11, were found to be exceedingly auspicious in terms of oil quality and yield performance. They also reported a negative and significant correlation between oleic and erucic acid, indicating the possibility of reducing the erucic acid content by enhancing oleic acid using an increased breeding intensity and potential donors. Sequence-tagged microsatellite site (STMS) analysis of canola variety ‘Heera’ and a high yielding popular one ‘Kranti’, clearly distinguished quality and non-quality lines. Seeds of interspecific hybrids obtained from the ovule culture of crosses between *B. junceia* and *B. napus* had a fatty acid profile different from parental values, mainly for oleic and erucic acids. The low oleic acid (13%) in *B. juncea* increased to 23–26% in hybrids, and high erucic acid in *B. juncea* (41%) declined to 21–23% among hybrids. Linoleic and linolenic acids showed slight variation from parental values. The fatty acid profile of F_1_ hybrids shifted towards that of canola quality. The F_2_ seeds had zero erucic acid and high oleic acid, similar to or exceeding the canola parent. Successful interspecific hybridization of *B. juncea* and *B. napus* was confirmed by altered FAP and molecular markers [18]. Low erucic varieties of *B. juncea* showed an increase in oleic and linoleic acid content, while double zero varieties of *B. napus* showed a proportional increase in oleic acid content and reduced erucic acid content [10]. In the present investigation, low erucic acid has been evidenced from R_0_ and R_1_ generations of putative somaclones of both genotypes. It occurred perhaps due to the metabolic pathway of erucic acid synthesis, as immature cotyledons and seeds have taken as explants sources, and plantlets were regenerated from cell lines derived from these explants.

### 2.4. Molecular Confirmation of Putative Drought Tolerant Plant (s)

**Figure 5:**
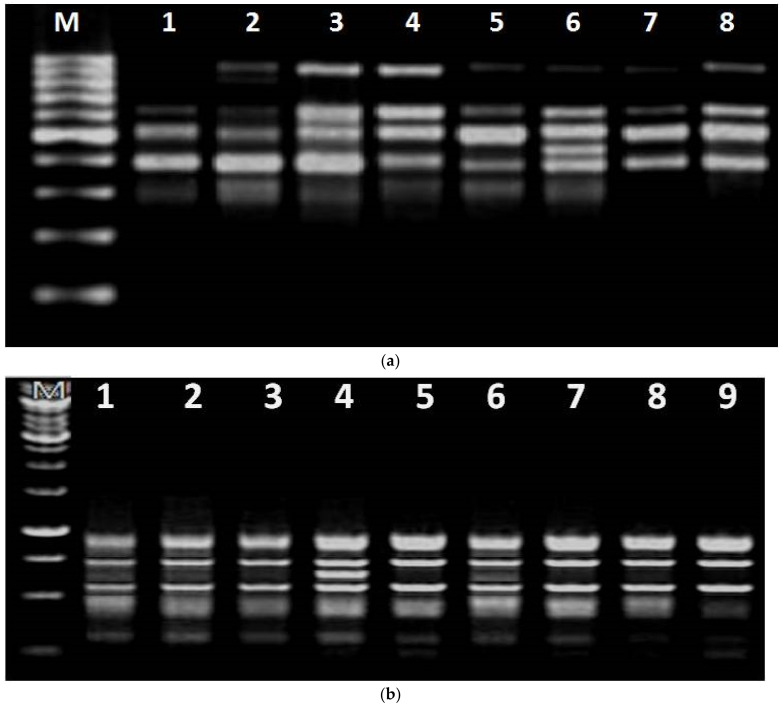
Electrophoretic banding pattern of the mother, as well as putative somaclone plants of (**a**) CS54, amplified with OPA-12 RAPD primer, Lane 1: Mother plant, Lane 2–8: Putative somaclones, M-1 kb ladder, (**b**) PM30, amplified with OPM-13 RAPD primer. Lane 1: Mother plant, Lane 2–9: Putative somaclones, M-1 kb ladder.

After hardening, seven plants of genotype CS54 and eight of genotype PM30 were subjected to molecular confirmation. A total of twenty-three RAPD markers (Table 9) were used to amplify mother plants and selected putative somaclones. Among all RAPD markers, OPA-12 was found to produce a polymorphic band (~900 bp) with template DNA of seven selected putative plants produced after considering CS54 as the mother genotype (Figure 5a). This band was seen in all the putative somaclones tested while absent in the mother plant. A unique band (~400 bp) was amplified in a selected somaclone plant and was absent in others. Consequently, primer OPM-13 amplified a polymorphic band (~350 bp) with a putative somaclone plant selected from PM30; however, the absence of this polymorphic band in the donor genotype confirms the presence of variability between the somaclones and mother plants (Figure 5b). Similarly, applications of RAPD markers for the same purposes have been reported by other researchers [25,45] also.

It has been proven that erucic acid content is governed by the fatty acid elongase 1 (*FAE1*) gene that translates the enzyme β-ketoacyl-CoA synthase (KCS) in erucic acid biosynthesis alleyway and catalyzes the first four enzymatic reactions in the synthesis of very-long-chain monounsaturated fatty acids (VLCMFAs) [1,2,46,47,48]. The mutation in the *FAE1* gene primes the forfeiture of occupation in enzymatic action and diminishes the gathering of VLCMFAs in seeds [1,2,49,50]. In the current research, plants with lower erucic acid have been obtained within R_0_ and R_1_ generations of putative somaclones raised from both the genotypes compared to mother plants, possibly due to mutations.

The present study is a probable first of its kind, regenerating low-erucic acid-containing variants employing a suspension culture for *Brassica juncea*. The conventional breeding scheme reports, limited success in procuring low erucic acid content variants. In an earlier attempt, Roy and Saha [17] screened low-erucic acid comprising genotypes of Indian mustard by another culture of the F1 hybrids. They identified three plants with erucic acid content lower than that of parental cultivars in the A_2_ generation. In the transgenic approach conducted by Sinha et al. [19], the fatty acid profile of the mature seed resulted in a 64–82% decrease in erucic acid production. The altered seed fatty acid compositions in transgenic lines showed significant increases in the levels of C18:1 and C16:0 or C18:0, along with enhancement in the ratio of C18:2/C18:3 and C18:1/C22:1. The reduction of C22 quantitatively accounted for the increase in the pool of C16 and C18 fatty acids in the seed oil developed by metabolic engineering involving both the plastidial and cytoplasmic enzyme approaches. Novel FatB genes were cloned and validated by Jha et al. [20] and showed potential application in metabolic engineering through their over-expression in seed tissues to generate stearate-rich vegetable fats/oils of commercial importance. Chaudhary et al. [6] offered mustard genotypes NRCM-120 and SKM-9033 with a low content of erucic acid and sinigrin, respectively, for their possible use in the development of a double zero variety. An attempt was also made to induce salt tolerance using in vitro production of *Brassica* [15,16].

Further, the environmental and nutritional conditions can be controlled uniformly and precisely under tissue culture conditions, and at a given time large number(s) of somatic cell lines could be screened swiftly to regenerate variants. In genus *Brassica*, the in vitro regeneration capabilities have been genotype-specific [31,51]. Levels of variations have been observed to be dependent on the regeneration potential of the genotypes. Earlier, similar variations have been identified in vitro by Shyam et al. [31] in *B. juncea,* Hachey et al. [52] in *B. campestris*, and by Zhang and Bhalla [53] in *B. napus*.

## 3. Materials and Methods

### 3.1. Experimental Materials

Two genotypes were selected based on different morpho-physiological, biochemical, and SSR molecular characterizations of Indian mustard genotypes [3,5,6,7]. These genotypes, namely CS54 and PM30, contained high and low erucic acid in their oil, respectively. Experimental materials were obtained from Zonal Agricultural Research Station, Morena, Rajmata Vijayaraje Scindia Agricultural University, Gwalior, India and AICRP on Rapeseed and Mustard, Indian Agricultural Research Institute, New Delhi, India. Fatty acid analysis was conducted at the Department of Botany, Banaras Hindu University, Varanasi, India. In order to explore low erucic acid content, callus and liquid cultures were established from immature cotyledons and seeds explant cultures of the above two genotypes with the assumption that immature seeds contain low erucic acid content in developmental phases and cell lines derived from these explants might have low erucic acid.

### 3.2. Culture Media

MS [54] was employed as a basal medium during the present course of investigations. In addition to MS basal micro and macro salts and vitamins, two diverse auxins (individually), viz., 2, 4-Dichlorophenoxyacetic acid (2,4-D) and NAA (α-Naphthalene acetic acid) and two varied cytokinins (singly), i.e., BAP (6-Benzyl adenine or 6-Benzyl amino purine) and TDZ (Thidiazuron) at different levels, as well as 2,4-D and NAA in association with BAP, 30.0 g L^−^^1^ sugar and 7.5 g L^−^^1^ agar, was supplemented to establish callus cultures from immature cotyledons and seeds, in turn, raising embryogenic cell suspension cultures. The culture media was prepared by making the final volume 1 liter, pH was adjusted to 5.8 ± 0.1 with 1N NaOH/HCl, and 7.5 g L^−^^1^ agar was added as a gelling agent. However, in liquid media for cell suspension culture, agar was not incorporated. The warm media in liquid state were dispensed into culture bottles (50–70 mL/bottle) and culture tubes (20–20 mL/tube) followed by sterilization at 121 °C and 15 psi pressure for 25–30 min in an automatic autoclave. Pre-sterilized 100 × 17 mm glass Petri dishes were used for the culture. Media combinations and other ingredients were shortlisted during preliminary experiments carried out in our laboratory. Basal media, PGR’s and other supplements were procured from Hi-Media^®^ Laboratories, Mumbai, India.

### 3.3. Establishment of Callus Cultures

Silique containing immature seeds were acquired and treated with Tween-20 for 10 min following cleaning with double distilled water followed by treatment with 0.25% (*w*/*v*) Carbendazim for 2 min followed by a rinsing with deionized water. The silique was further exposed to 70% ethyl alcohol for 2 min followed by surface sterilization by immersing in 0.1% mercuric chloride (HgCl_2_) for 1–2 min and finally three times with sterilized distilled water. Immature cotyledon (0.1 cm) and 1 to 2 mm sized immature seeds were excised from siliquae and cultured on pre-sterilized 100 × 17 mm glass Petri dishes containing 25–30 mL/dish media. All the cultures were incubated in racks inside a culture room exposed to a photoperiodic cycle of 16 h, at an intensity of 1600 lux luminance provided with cool white fluorescent light and 8h darkness at 25 ± 2 °C and 70% RH.

### 3.4. Establishment of Embryogenic Cell Suspension Culture

Embryogenic suspension cultures were started by conveying ~2.0 g embryogenic calli of 6 to 8 weeks old acquired from immature cotyledons and seeds cultures to 250 mL Erlenmeyer flasks holding 50 mL MS fluid medium. Callus pieces were strained through a stainless-steel mesh (1 mm) and were disquieted on a horizontal shaker (120 rpm) at 25 ± 2 °C in the absolute dark. After two weeks, cultures were subjected, under a photoperiod regime, each of 12 h light and dark at an intensity of 1200 lux luminance provided by white, fluorescent light. After two weeks, the cultures were sieved aseptically to eliminate large clumps, and 10 mL filtrate was appended with 40 mL of a fresh medium. The leftover filtrate was subcultured to establish new suspension cultures and for regeneration.

### 3.5. Maintenance and Regeneration of Putative Somaclones from Cell Lines

Callus cultures/somatic embryoids of two genotypes viz., CS54 and PM30, were obtained from callus cultures. They sustained through continuous sub-culturing and were cut into small pieces. Five pieces of callus and 10–20 cell clumps acquired from liquid culture were picked arbitrarily and inoculated in a Petri dish containing selective media MS.5B (MS + 0.5 mg L^−^^1^ BAP) for regeneration and on non-selective media MS.5D.5B (MS + 0.5 mg L^−^^1^ 2,4-D + 0.5 mg L^−^^1^ BAP) or MSN.5B (MS +1.0 mg L^−^^1^ NAA + 0.5 mg L^−^^1^ BAP) for the continuation of organogenetic calli growth. After one month of incubation on the selection medium, callus portions with green shoot primordia were separated and transferred into shoot regeneration medium MS.5B (MS + 0.5 mg L^−^^1^ BAP). Well-developed cultures with regenerated shootlets were divided into pieces after 45 days and subcultured once again on regeneration media in glass jars to obtain multiple shoots and growth. The shootlets were allowed to grow under growth room conditions at 25 ± 2 °C, RH 75% and 1600 lux for 16 h light and 8 h dark cycles. Well-developed normal-looking shootlets of appropriate length (<7.5 cm) were then transferred to the rooting MS medium amended with 0.5 mg L^−^^1^ indole-3-butyric acid (IBA), 15.0 g L^−^^1^ sucrose, and 7.5 g L^−^^1^ agar in line with the suggestion of Tripathi et al. [38] until ample roots were developed.

Shootlets with roots were designated as putative Somaclone-1 and Somaclone-2, regenerated from cell lines of genotypes CS54 and PM30, respectively. After rooting, plantlets with sufficient roots were excised from the medium carefully, rinsed systematically until the entire agar media was removed from the plantlet′s surface. The rooted regenerants were then subjected to the nursery pot supplied vermiculate, farmyard manure (FYM) and sand (1:1:1) for 30 days, maintaining the light, humidity, and temperature at greenhouse conditions. After 30 days, well-developed regenerants were placed in a net house for 45 days before being transferred to field conditions, where they were grown till maturity and seed set.

### 3.6. Morpho-Physiological Evaluation of Regenerants

Regenerants were assumed as putative somaclones, and R_0_ and R_1_ generations were compared with their respective mother plants for different morpho-physiological parameters viz., plant height, number(s) of primary and secondary branches, days to 50% flowering and maturity, number(s) of siliqua per plant, siliqua length, number(s) of seed per siliqua, seed yield per plant, and biological yield during field evaluation.

### 3.7. In Vivo Testing of Regenerants for Fatty Acids Profiling

The plantlets regenerated from in vitro cultures subjected to fatty acids analysis to characterize and quantify different fatty acids, including erucic acid content.

### 3.8. Procedure for Preparation of Methyl Esters for Fatty Acid Profiling

Five grams of Brassica seeds of both the genotypes were ground with a pestle and mortar to obtain a fine powder. The powder was shifted to a completely dry test tube (50 mL capacity, 25 *×* 150 mm), and 5 mL methanol was added to each tube, followed by two drops of concentrated H_2_SO_4_. Tubes containing oil–methanol–acid blends were kept in a water bath at 65 °C for an hour. Then tubes were cooled to room temperature, followed by the addition of 2.0 mL hexane into each tube. The tubes were vortexed and held till the hexane layer was settled out that contains methyl esters. The hexane layer (1.0–1.5 mL) was removed carefully using a micropipette and subjected to a 2 mL screw-capped tube. A small amount of hydrous sodium sulfate was added to each tube to seize moisture. A total of 1 mL of the hexane layer holding methyl esters was injected into a pre-conditioned gas chromatograph (GC).

The diverse fatty acids were sorted by their relative retention times and equivalencing with identified standards. The percent composition of fatty acid was decided by calculating the area under each peak. Fatty acids were analyzed by applying gas-liquid chromatography (Perkin Elmer Clarus 500) fitted with a mega bore column (30 m long and 0.53 mm Ø) assembled with OV-101, methyl silicone polymer, and applying a FID (flame ionization detector). The column temperature (150–270 °C), injector temperature (250 °C), and detector temperature (250 °C) were maintained. GLC was set to 10 °C per minute increment, and at the end, it was kept at 270 °C.

### 3.9. Molecular Confirmation of Putative Somaclones Using RAPD Markers

DNA was extracted from the young leaves of genotypes, viz., CS54 and PM30, and selected putative somaclone plants using the Qiagen DNA extraction kit. Extracted DNA samples were quantified with the help of a nanodrop spectrophotometer and diluted up to 25 ng/µL. For PCR amplification, a total of 23 random decamer primers (Table 9) were employed. The PCR reaction mixture comprised 50 ng genomic DNA, 10 pmol primer, 200 μM of each dNTP, and 1 unit of *Taq* DNA polymerase with PCR buffer (Tris HCl, pH 9.0; 15 mM MgCl_2_) added. The cycling parameters were: 45 cycles at 94 °C of 1 min, 1 min at 36 °C, 2 min at 72 °C with a final extension time of 7 min at 72 °C. Amplicons were separated by electrophoresis on 1.4% agarose gel and envisaged under a gel documentation system after staining with ethidium bromide.

### 3.10. Experimental Design and Data Analysis

Experiments on in vitro culture, cell suspension culture, and plantlet regeneration were conducted in CRD (completely randomized design) with two replications for each treatment. For each replication, around 200 explants/cell clumps/embryoids were plated per culture media treatment. Fatty acid profiling was also conducted in CRD in two replications. While morpho-physiological data were analyzed in a randomized block design (RBD) with three replications and the data were analyzed as suggested by Snedecor and Cochran [55]. The significant difference between different treatments was observed by Duncan’s multiple range test (DMRT) at *p* < 0.05. The same letters in one treatment represent non-significant differences at *p* < 0.05.

## 4. Conclusions

In conclusion, two putative somaclones with low erucic acid derived from the cell lines of genotypes CS54 and PM30 have been developed during in vitro culturing, providing a reliable source of variability. Future studies must understand the mechanism of variable erucic acid content in somaclones derived from in vitro cultures, especially suspension cultures. It will help decide the selection strategy for developing low-erucic acid varieties of Indian mustard toward breeding and biotechnological prospects. These putative somaclones may be used in molecular breeding programs, considering them as a parent in the crop improvement program.

## Figures and Tables

**Table 1:** Callus induction, formation of morphogenic calli and plantlet regeneration efficiency from cultured immature cotyledons.

S. No.	Culture Medium	MS3D (MS + 3.0 mg L^−1^ 2,4-D) Medium		MS.5B (MS + 0.5 mg L^−1^ BAP) Medium			
	Genotype	Callus Induction (%) *	Callus Features	Morphogenic Calli (%) *	Shoot Regeneration * (%)	Average Number(s) of Shoots/Explants *	Mean Shoot Length *
1.	CS54	72.47 ^a^ ± 1.31	Dark brown and friable	68.40 ^a^ ± 0.82	62.32 ^a^ ± 0.66	7.98 ^a^ ± 0.16	6.27 ^a^ ± 0.12
2.	PM30	77.83 ^b^ ± 1.45	Cream and light compact	74.68 ^b^ ± 0.88	69.77 ^b^ ± 0.72	8.75 ^b^ ± 0.18	7.43 ^b^ ± 0.15
CD_0.05_		2.794		3.85	3.59	0.375	0.875

Data was analyzed in CRD with two replications. * Mean ± standard deviation. ^a,b^ Values within the column followed by different letters are significantly different, and the same letters are not different at a 5% probability level by Duncan’s multiple range test.

**Table 2:** Callus induction, formation of morphogenic calli and plantlet regeneration efficiency from cultured immature seeds.

S. No.	Culture Medium	MS3D (MS + 3.0 mg L^−1^ 2,4-D) Medium		MS.5B (MS + 0.5 mg L^−1^ BAP) Medium			
	Genotype	Callus Induction (%) *	Callus Features	Morphogenic Calli (%) *	Shoot Regeneration (%) *	Average Number(s) of Shoots/Explants *	Mean Shoot Length *
1.	CS54	81.80 ^a^ ± 1.76	Dark brown and friable	76.60 ^a^ ± 1.26	72.45 ^a^ ± 0.65	8.23 ^a^ ± 0.16	6.57 ^a^ ± 0.17
2.	PM30	86.42 ^b^ ± 1.83	Cream and light compact	84.42 ^b^ ± 1.37	78.89 ^a^ ± 0.73	9.45 ^b^ ± 0.22	7.54 ^b^ ± 0.19
CD_0.05_		2.95		2.632	2.721	0.250	0.301

Data was analyzed in CRD with two replications. * Mean ± standard deviation. ^a,b^ Values within column followed by different letters are significantly different, and the same letters are not different at a 5% probability level by Duncan’s multiple range test.

**Table 3:** Establishment of embryogenic cell suspension cultures and plantlet regeneration from callus raised from immature seeds and cotyledons explants.

S. No.	Genotypes	Initiation of Embryogenic Cell Suspension Culture on MS3D.5B (MS + 3.0 mg L^−1^ 2,4-D + 0.5 mg L^−1^ BAP) Liquid Medium		Shoot Regeneration (%) on MS.5D.5B (MS + 0.5 mg L^−1^ 2,4-D + 0.5 mg L^−1^ BAP) *	Shoot Regeneration (%) on MS.5N.5B (MS + 0.5 mg L^−1^ NAA + 0.5 mg L^−1^ BAP) *
		Increment in Fresh Weight (FW in g) *	Relative Growth Rate (RG) in % *		
1.	CS54	5.72 ^a^ ± 0.34	186 ^a^ ± 3.86	34.34 ^a^ ± 0.56	67.50 ^a^ ± 0.84
2.	PM30	6.30 ^b^ ± 0.42	215 ^b^ ± 4.47	38.78 ^b^ ± 0.73	76.60 ^b^ ± 0.92
	CD_0.05_	0.540	6.731	1.537	2.399

Initial fresh weight was taken as 2.0 g friable callus per flask containing 50 mL liquid media. The evaluation was made after 45 days in culture FW: Fresh weight; RG: Relative growth. Data was analyzed in CRD with two replications. * Mean ± standard deviation. ^a,b^ Values within the column followed by different letters are significantly different, and the same letters are not different at a 5% probability level by Duncan’s multiple range test.

**Table 4:** Comparison of morpho-physiological parameters between mother plants, R_0_, and R_1_ generations of putative somaclones * regenerated from cell lines of genotypes CS 54 and PM30.

S. No.	Genotype	CS54			CD_0.05_	PM30			CD_0.05_
	Parameters	Mother Plant	Putative Somaclone (R_0_) Generation	Putative Somaclone (R_1_) Generation		Mother Plant	Putative Somaclone (R_0_) Generation	Putative Somaclone (R_1_) Generation	
1.	Plant height (cm)	150.0 ^b^ ± 2.52	115.0 ^a^ ± 1.50	117 ^a^ ± 1.54	4.71	145.0 ^b^ ± 2.32	90.0 ^a^ ± 1.52	93.0 ^a^ ± 1.54	3.21
2.	Number(s) of primary branches	4.0 ^a^ ± 0.53	4.0 ^a^ ± 0.56	4.2 ^a^ ± 0.58	0.74	6.0 ^a^ ± 0.54	6.0 ^a^ ± 0.52	6.12 ^a^ ± 0.62	1.01
3.	Number(s) of secondary branches	7.0 ^a^ ± 0.64	8.0 ^b^ ± 0.64	8.44 ^b^ ± 0.70	0.76	6.0 ^a^ ± 0.42	8.0 ^b^ ± 0.48	7.90 ^b^ ± 0.44	0.89
4.	Days to 50% flowering	30.0 ^a^ ± 2.08	28.0 ^a^ ± 2.02	27.34 ^a^ ± 2.11	2.54	35.0 ^a^ ± 2.08	34.0 ^a^ ± 1.52	36.0 ^a^ ± 1.88	3.42
5.	Days to maturity	128.0 ^b^ ± 2.65	100.0 ^a^ ± 2.08	102 ^a^ ± 2.05	2.72	130.0 ^b^ ± 2.0	115.0 ^a^ ± 1.62	118.0 ^a^ ± 1.64	3.27
6.	Siliqua length	5.7 ^b^ ± 0.42	5.0 ^a^ ± 0.38	3.98 ^a^ ± 0.36	1.04	5.6 ^b^ ± 0.40	4.3 ^a^ ± 0.32	4.4 ^a^ ± 0.34	1.05
7.	Number(s) of siliqua per plant	200 ^c^.0 ± 3.06	130.0 ^a^ ± 2.21	135 ^b^ ± 2.88	3.66	137.0 ^a^ ± 3.06	140.0 ^a^ ± 2.54	138.0 ^a^ ± 2.08	3.83
8.	Number(s) of seed per siliqua	12.0 ^b^ ± 0.98	8.0 ^a^ ± 0.64	8.62 ^a^ ± 0.89	1.17	13.0 ^b^ ± 1.10	7.0 ^a^ ± 0.90	7.22.0 ^a^ ± 1.08	2.65
9.	Number (s) seeds per plant	2400.0 ^b^ ± 4.3	1040.0 ^a^ ± 3.21	1044.0 ^a^ ± 3.30	5.38	1781.0 ^b^ ± 4.04	840.0 ^a^ ± 3.61	845.0 ^a^ ± 3.82	5.97
10.	Seed yield per plant (g)	4.6 ^b^ ± 0.44	1.90 ^a^ ± 0.30	1.98 ^a^ ± 0.35	0.84	3.8 ^b^ ± 0.45	1.6 ^a^ ± 0.25	1.8 ^a^ ± 0.34	0.79
11.	Biological yield per plant (g)	26.0 ^b^ ± 1.28	13.0 ^a^ ± 0.52	14.0 ^a^ ± 0.56	1.12	24.0 ^b^ ± 1.13	20.86 ^a^ ± 1.46	22.0 ^a^ ± 1.38	1.61

* Putative somaclones were transferred in mid-December 2018 for 15 days under greenhouse conditions (temperature 280C, RH 70%, and photoperiod regimes 16 h light and 8 h dark). Putative somaclones (R_0_ generation) were transferred in January 2019 under field conditions. R_1_ generation was planted in November 2019 under field conditions. Morpho-physiological observations were recorded after 60 days of putative somaclone regeneration from cell lines of genotype CS-54. The morphological observation was recorded after 75 days of putative somaclone regeneration from cell lines of genotype PM30. The experiment was conducted in randomized block design. * Mean ± standard deviation. ^a,b^ Values within the column followed by different letters are significantly different, and the same letters are not different at a 5% probability level by Duncan’s multiple range test.

**Table 5:** Fatty acid profiling of putative somaclone regenerated from cell lines of genotype CS54 using GC-MS.

Group No. According to RT	Components	Compound Characterized	Formula	Molar Weight	Apex RT	Start RT	End RT	Area	% Area	Height	% Height	Probability
1	1	1-Decanol, 2-octyl-	C_20_H_42_O	298.55	5.24	5.16	5.35	3 × 10^7^	4.01	7 × 10^6^	7.11	3.54
	2	Heptafluorobutyric acid, n-octadecyl ester	C_22_H_37_F_7_O_2_	466.52								3.40
	3	1-Dodecanol, 2-hexyl	C_16_H_34_O	242.44								3.27
2	1	Hexadecanoic acid, methyl ester	C_18_H_36_O_2_	284.50	7.4	7.23	7.68	6 × 10^7^	9.30	1 × 10^7^	10.72	68.41
	2	Pentadecanoic acid, 13-methyl-, methyl ester	C_17_H_34_O_2_	270.50								12.82
	3	Pentadecanoic acid, 14-methyl-, methyl ester	C_17_H_34_O_2_	270.50								2.77
3	1	2,5-Furandione, dihydro-3-isooctadecyl-	C_22_H_40_ O_3_	352.55	9.66	9.59	10.04	4 × 10^7^	6.69	5 × 10^6^	4.55	7.43
	2	L-Serinamide,1-methyl-5-oxo-L-prolyl-N,1-dimethyl-L-his tidyl-N,1-L-tryptophyl-N,N,N2,O-tetramethyl-	C_34_H_48_N_8_O_6_	664.79								5.10
	3	4,6-Dipropyl-nonan-5-one	C_15_H_30_O	226.40								3.39
	4	Ethyl iso-allocholate	C_26_H_44_O_5_	436.62								26.54
	5	7,8-Epoxylanostan-11-ol, 3-acetoxy-	C_32_H_54_O_4_	502.82								17.67
	6	Docosanoic acid, 1,2,3-propanetriyl ester	C_69_H_134_O_6_	1059.79								4.81
4	1	Ethy 9,12,15-octadecatrienoate	C_20_H_34_O_2_	306.48	10.4	10.23	10.68	7 × 10^7^	10.9	1 × 10^7^	11.31	10.24
	2	9,12,15-Octadecatrienoic acid, methyl ester, (Z,Z,Z)-	C_19_H_32_O_2_	292.50								8.65
	3	N-Propy1 9,12,15-octadecatrienoate	C_21_H_36_O_2_	320.50								6.80
5	1	Ethyl 9,12,15-octadecatrienoate	C_20_H_34_O_2_	306.48	11.4	11.27	11.65	2 × 10^7^	2.88	2 × 10^6^	1.83	10.24
	2	9,12,15-Octadecatrienoic acid, methyl ester, (Z,Z,Z)-	C_19_H_32_O_2_	292.50								8.65
	3	N-Propyl 9,12,15-octadecatrienoate	C_21_H_36_O_2_	320.56								6.80
6	1	Methyl 11-docosenoate	C_23_H_44_O_2_	352.60	15.33	15.17	15.65	3 × 10^7^	5.48	5 × 10^6^	5.1	49.90
	2	13-Docosenoic acid, methyl ester, (Z)-	C_23_H_44_O_2_	352.59								13.33
	3	Cis-13-Docosenoyl chloride	C_22_H_41_ClO	357.00								12.29
7	1	Ethyl iso-allocholate	C_26_H_44_O_5_	436.62	17.05	16.93	17.39	6 × 10^7^	9.49	8 × 10^6^	7.64	11.95
	2	(5á)Pregnane-3,20á-diol,14à,18à-[4-methyl-3-oxo-(1-oxa-4-azabutane-1,4-diyl)]-, diacetate	C_21_H_36_O_2_	320.50								7.48
	3	4H-Cyclopropa [5’,6’]benz[1 ‘,2’:7,8]azuleno[5,6-b]oxiren-4-one,8,8a-bis(acetyloxy)-2a-[(acetyloxy)methyl]-1,1a,1b,1c,2a,3,3a,6a,6b,7,8,8a-dodecahydro-6b-Hydroxy-3a-methoxy-1,1,5,7-tetramethyl-,[1aR-(1aà,1bá,1cà,2aà,3aà,6aà,6bà,7à,8á,8aà)]-	C_26_H_34_O_11_	522.54								5.13
	4	17-(1,5-Dimethylhexyl)-2,3-dihydroxy-10,13-Dimethyl-1,2,3,7,8,9,10,11,12,13,14,15,16,17-tetradecahydrocyclopenta[a]phenanthren-6-One	C_29_H_50_O	414.71								21.34
	5	Propanoic acid, 2-(3-acetoxy-4,4,14-trimethylandrost-8-en-17-yl)-	C_27_H_42_O_4_	430.6								14.64
	6	2,4a-Oxymethano-1,2,3,4,4a,4b,5,6,7,8,8a,9-d odecahydrophenanthren-9-one,8-cyanomethyl-2-methoxy-7-methoxycarbon yl-1,1,7-trimethyl-	C_20_H_28_O_5_	348.433								14.07
8	1	Glycidyl oleate	C_21_H_38_O_3_	338.50	21.09	20.97	21.33	4 × 10^7^	6.05	6 × 10^6^	5.6	71.7
	2	9-Octadecenoic acid, 1,2,3-propanetriyl ester,(E,E,E)-	C_57_H_104_O_6_	885.43								4.87
	3	9-Octadecenoic acid (Z)-,2-hydroxy-1-(hydroxymethyl)ethyl ester										4.68
9	1	9,12,15-Octadecatrienoic acid,2-phenyl-1,3-dioxan-5-yl ester	C_28_H_40_O_4_	440.00	23.73	23.62	23.9	2 × 10^7^	3.68	3 × 10^6^	3.09	13.64
	2	Methyl 2-hydroxy-octadeca-9,12,15-trienoate	C_19_H_32_O_3_	308.50								3.94
	3	Butyl 6,9,12,15-octadecatetraenoate										3.78
10	1	Glycidyl oleate	C_21_H_38_O_3_	338.52	27.61	27.56	27.75	2 × 10^7^	3.17	4 × 10^6^	3.91	34.58
	2	2,3-Dihydroxypropyl cis-13-docosenoate	C_25_H_48_	412.64								6.97
	3	9-Octadecenoic acid, 1,2,3-propanetriyl ester,(E,E,E)-	C_57_H_104_O_6_	885.43								3.8
11	1	E,E,Z-1,3,12-Nonadecatriene-5,14-diol	C_19_H_34_O_2_	294.47	32.19	32.06	32.37	3 × 10^7^	4.77	4 × 10^6^	3.92	7.41
	2	Trilinolein	C_57_H_98_O_6_	879.40								4.78
	3	Tricyclo[20.8.0.0(7,16)]triacontane,1(22),7(16)-diepoxy-	C_30_H_52_O_2_	444.73								4.23

**Table 6:** Comparison of fatty acid composition between mother plants and R_0_ and R_1_ generations of putative somaclones regenerated from cell lines of genotypes CS 54.

S. No.	Biochemical Parameters	Formula	Molar Weight	Mother Plant (%)	Biochemical Parameters Detected in Somaclone	Apex RT	Value (%) in CS54 Soma Clone (R_0)_ Generation Or % Area	Value (%) in Soma Clone CS54 (R_1)_ Generation Or % Area	CD_0.05_
1	Palmitic acid (%)	C_16_H_32_O_2_	256.42	5.91 ^a^ ± 0.16 *	Hexadecanoic acid, methyl ester	7.4	9.30 ^b^ ± 0.20 *	9.21 ^b^ ± 0.22 *	0.32
					Pentadecanoic acid, 13-methyl-, methyl ester				
					Pentadecanoic acid, 14-methyl-, methyl ester				
2	Oleic acid (%)	C_18_H_34_O_2_	282.461	12.89 ^b^ ± 0.28 *	Glycidyl oleate	21.09	6.05	6.01	0.39
					9-Octadecenoic acid, 1,2,3-propanetriyl ester, (E, E, E)-				
					9-Octadecenoic acid (Z)-,2-hydroxy-1-(hydroxymethyl) ethyl ester				
					Glycidyl oleate	27.61	3.17	3.30	
					2,3-Dihydroxypropyl cis-13-docosenoate				
					9-Octadecenoic acid, 1,2,3-propanetriyl ester, (E, E, E)-				
Total							9.22 ^a^ ± 0.24 *	9.31 ^a^ ± 0.26 *	
3	Linoleic acid (%)	C_18_H_32_O_2_	280.40	2.96 ± 0.11 *	-	-	-	-	-
4	Linolenic acid (%)	C_18_H_30_O_2_	278.40	9.41 ^a^ ± 0.18 *	Ethy 9,12,15-octadecatrienoate	10.4	10.90	10.78	
					9,12,15-Octadecatrienoic acid, methyl ester, (Z, Z, Z)-				
					N-Propy1 9,12,15-octadecatrienoate				
					Ethyl 9,12,15-octadecatrienoate	11.4	2.88	2.94	
					9,12,15-Octadecatrienoic acid, methyl ester, (Z, Z, Z)-				
					N-Propyl 9,12,15-octadecatrienoate				
					9,12,15-Octadecatrienoic acid,2-phenyl-1,3-dioxan-5-yl ester	23.73	3.68	3.70	
					Methyl 2-hydroxy-octadeca-9,12,15-trienoate				
					Butyl 6,9,12,15-octadecatetraenoate				
Total							17.46 ^b^ ± 0.25 *	17.42 ^b^ ± 0.28 *	0.420
5	Erucic acid (%)	C_22_H_42_O_2_	338.6	41.36 ^b^ ± 0.44 *	Methyl11-docosenoate13-Docosenoic acid, methyl ester, (Z)- Cis-13-Docosenoyl chloride	15.33	5.48 ^a^ ± 0.098 *	5.52 ^a^ ± 0.10 *	0.53

* Mean ± standard deviation. ^a,b^ Values within the column followed by different letters are significantly different, and the same letters are not different at a 5% probability level by Duncan’s multiple range test.

**Table 7:** Fatty acid profiling of putative somaclone regenerated from cell lines of genotype PM30 using GC-MS.

Group No. According to RT	Components	Compound Characterized	Formula	Molar Weight	Apex RT	Start RT	End RT	Area	% Area	Height	% Height	Probability
1	1	1-Octadecene	C_18_H_36_	252.50	5.24	5.19	5.32	2.14 × 10^8^	2.71	79,764,789	10	3.6
	2	E-15-Heptadecenal	C_17_H_32_O	252.43								3.32
	3	Nonacos-1-ene	C_29_H_58_	406.80								3.06
2	1	Hexadecanoic acid, methyl ester	C_17_H_34_O_2_	270.45	7.42	7.16	7.91	7.67 × 10^8^	9.72	75,578,774	9.47	78.51
	2	Pentadecanoic acid, 13-methyl-, methyl ester	C_17_H_34_O_2_	270.45								9.57
	3	Hexadecanoic acid, 2-methyl-	C_17_H_34_O_2_	270.50								2.76
3	1	9-Octadecenoic acid (Z)-, methyl ester	C_19_H_36_O_2_	296.50	9.79	9.64	10.21	9.05 × 10^8^	11.46	87,487,645	10.96	15.76
	2	Cis-13-Octadecenoic acid, methyl ester	C_19_H_36_O_2_	296.50								11.75
	3	Trans-13-Octadecenoic acid, methyl ester	C_19_H_36_O_2_	296.48								10.38
4	1	1-Heptacosanol	C_27_H_56_O	396.73	11.99	11.86	12.25	1.28 × 10^8^	1.62	13,276,684	1.66	3.86
	2	Hexacosylpentafluoropropionate	C_29_H_53_F_5_O_2_	528.70								3.26
	3	Hexacosyl heptafluorobutyrate	C_30_H_53_F_7_O_2_	578.70								3.14
	4	17-Pentatriacontene	C_35_H70	490.90								21.44
	5	Oleic acid, 3-(octadecyloxy)propyl ester	C_39_H_76_O_3_	593.00								14.27
	6	Octadecane, 3-ethyl-5-(2-ethylbutyl)-	C_26_H_54_	366.70								11.22
5	1	Glycidyl palmitate	C_19_H_36_O_3_	312.48	17.13	17.06	17.35	1.66 × 10^8^	2.11	27,307,391	3.42	86.71
	2	Hexadecanoic acid,2-hydroxy-1-(hydroxymethyl)ethyl ester	C_19_H_38_O_4_	330.50								2.04
	3	Hexadecanoic acid,1-(hydroxymethyl)-1,2-ethanediyl ester	C_19_H_38_O_4_	330.50								1.48
6	1	Glycidyl oleate	C_21_H_38_O_3_	338.50	21.39	21.05	22.24	2 × 10^9^	25.36	98,040,231	12.29	89.2
	2	9-Octadecenoic acid (Z)-,2-hydroxy-1-(hydroxymethyl)ethyl ester	C_21_H_38_O_4_	354.52								2.17
	3	9-Octadecenoic acid, 1,2,3-propanetriyl ester,(E,E,E)-	C_57_H_10__4_O_6_	885.43								1.62
7	1	Phenol, 2,4-bis(1,1-dimethylethyl)-,phosphite (3:1)	C_42_H_63_O_3_P	646.92	28.27	27.71	28.48	3.4 × 10^8^	4.31	28,193,887	3.53	90.45
	2	Silane, diethylheptyloxyoctadecyloxy-	C_29_H_62_O_2_Si	470.90								2.2
	3	Methylenebis(2,4,6-triisopropylphenylphosphine)	C_31_H_50_P	484.67								1.55
	4	Phenol, 2,4-bis(1,1-dimethylethyl)-,phosphite (3:1)	C_42_H_63_O_3_P	646.92								47.29
	5	1-Cholestanone, O-allyloxime	C_30_H_51_NO	441.70								8.23

**Table 8:** Comparison of fatty acid composition between mother plants and R_0_ and R_1_ generations of putative somaclones regenerated from cell lines of genotypes PM30.

S. No.	Biochemical Parameters	Mother Plant (%)	Other Names of Biochemical Parameters	Apex RT	Value (%) in PM 30 Soma Clone (R_0_) Generation Or % Area	Value (%) in PM 30 Soma Clone (R_1_) Generation Or % Area	CD_0.05_
1	Palmitic acid (%)	5.5 ^a^ ± 0.09 *	Hexadecanoic acid, methyl ester	7.42	9.72	9.84	0.236
			Pentadecanoic acid, 13-methyl-, methyl ester				
			Hexadecanoic acid, 2-methyl-				
			Glycidyl palmitate	17.13	2.11	2.06	
			Hexadecanoic acid,2-hydroxy-1-(hydroxymethyl) ethyl ester				
			Hexadecanoic acid,1-(hydroxymethyl)-1,2-ethanediyl ester				
Total					11.83 ^b^ ± 0.12 *	11.90 ^b^ ± 0.13 *	
2	Oleic acid (%)	25.6 ^a^ ± 0.51 *	9-Octadecenoic acid (Z)-, methyl ester	9.79	11.46	11.86	0.674
			Cis-13-Octadecenoic acid, methyl ester				
			Trans-13-Octadecenoic acid, methyl ester				
			1-Heptacosanol	11.99	1.62	1.40	
			Hexacosylpentafluoropropionate				
			Hexacosyl heptafluorobutyrate				
			17-Pentatriacontene				
			Oleic acid, 3-(octadecyloxy) propyl ester				
			Octadecane, 3-ethyl-5-(2-ethylbutyl)-				
			Glycidyl oleate	21.39	25.36	25.11	
			9-Octadecenoic acid (Z)-,2-hydroxy-1-(hydroxymethyl) ethyl ester				
			9-Octadecenoic acid, 1,2,3-propanetriyl ester, (E, E, E)-				
Total					38.44 ^b^ ± 0.59 *	38.37 ^b^ ± 0.62 *	
3	Linoleic acid (%)	2.75 ± 0.11 *	-	-	-	-	-
4	Linolenic acid (%)	15.55 ± 0.46 *	-	-	-	-	-
5	Erucic acid (%)	1.075 ± 0.015 *	-	-	-	-	-

* Mean ± standard deviation. ^a,b^ Values within the column followed by different letters are significantly different, and the same letters are not different ata 5% probability level by Duncan’s multiple range test.

**Table 9:** Details of random amplified polymorphic DNA (RAPD) primers used for the confirmation of the difference between mother plants and putative somaclones in the present study.

Primer	Sequence 5′-3′	CS54		PM30	
		Total Bands	Polymorphic Bands	Total Bands	Polymorphic Bands
OPE-09	CTTCACCCGA	4	0	7	0
OPE-17	CTACTGCCGT	5	0	4	0
OPF-03	CCTGATCACC	3	0	5	0
OPA-5	AGGGGTCTTG	4	0	4	0
OPA-8	GTGACGTAGG	5	0	6	0
OPC-10	TGTCTGGGTG	6	0	5	0
OPC-15	GACGGATCAG	5	0	4	0
OPAP-07	ACCACCCGCT	4	0	4	0
OPAP-13	TGAAGCCCCT	4	0	3	0
OPR-15	GGACAACGAG	5	0	4	0
OPM-05	GGGAACGTGT	3	0	5	0
OPM-12	CTGGGCAACT	4	0	4	0
OPM-13	GGTGGTCAAG	6	0	8	2
OPO-20	ACACACGCTG	4	0	2	0
OPB-18	CCACAGCAGT	5	0	4	0
OPE-06	AAGACCCCTC	4	0	4	0
OPE-15	ACGCACAACC	3	0	5	0
OPH-05	AGTCGTCCCC	3	0	4	0
OPH-14	ACCAGGTTGG	4	0	4	0
OPI-02	GGAGGAGAGG	5	0	6	0
OPI-08	TTTGCCCGGT	4	0	3	0
OPL-06	GAGGGAAGAG	3	0	3	0
OPO-10	TCAGAGCGCC	5	0	4	0
OPO-11	GACAGGAGGT	3	0	4	0
OPP-17	TGACCCGCCT	4	0	3	0
OPA-12	TCGGCGATAG	7	2	5	0

## Data Availability

The data presented in this study is available within this article.

## Abbreviations

MS	Murashige and Skoog
2,4-D	2,4 dichlorophenoxyacitic acid
BA-6	benzyladenine
NAA	α-naphthaleneacetic acid
IBA	indole-3-butyric acid
